# Effects of Different Temperatures on Flavonoid Secondary Metabolites and Antioxidant Activities of *Paecilomyces hepiali*

**DOI:** 10.3390/antiox15070892

**Published:** 2026-07-19

**Authors:** Bing Jia, Chuyu Tang, Haoxu Tang, Yan Tong, Jinxuan Yan, Yuling Li, Xiuzhang Li

**Affiliations:** State Key Laboratory of Plateau Ecology and Agriculture, Qinghai Academy of Animal and Veterinary Sciences, Qinghai University, Xining 810016, China; bingjia0415@163.com (B.J.); chuyutang0410@163.com (C.T.); haoxutang0717@163.com (H.T.); tongyan718@126.com (Y.T.); yanjinxuan2666@163.com (J.Y.)

**Keywords:** *Paecilomyces hepiali*, temperature, antioxidant capacity, metabolomics, flavonoids

## Abstract

To explore the physiological and metabolic response mechanism of *Paecilomyces hepiali (P. hepiali)* under varying temperatures, five temperature gradients were established in this study. Combined with in vitro antioxidant assays and untargeted and targeted metabolomics analyses, the variations in antioxidant system and flavonoid secondary metabolites were investigated. Results showed that temperature significantly affected the accumulation of active substances and antioxidant capacity, including of peroxidase (POD), total flavonoids, soluble protein and 2,2′-azino-bis (3-ethylbenzothiazoline-6-sulfonic acid) (ABTS). Metabolomics analysis revealed distinct metabolic profiles among groups, and flavonoids and phenolic acids were identified as pivotal responsive metabolites. KEGG enrichment analysis revealed that flavonoid biosynthesis, linoleic acid metabolism and arachidonic acid metabolism served as core regulatory pathways. Nineteen common differential flavonoid metabolites were screened out. Correlation analysis revealed that vanillin, cryptochlorogenic acid and sinapinic acid strongly correlated with antioxidant capacity, acting as key regulators for temperature-induced antioxidant responses in *P. hepiali*. This study verified that temperature modulates the antioxidant system and flavonoid metabolism of *P. hepiali*, offering references for industrial optimization of its fermentation.

## 1. Introduction

Chinese cordyceps, a rare and precious traditional Chinese medicinal material endemic to the Qinghai–Tibet Plateau, is a complex composed of the stroma and insect larvae formed by the parasitism of Chinese cordyceps fungus on the larvae of Hepialidae insects [1,2]. Studies have shown that the Chinese cordyceps microbial community contains a variety of symbiotic and associated fungi [3,4], among which *Paecilomyces hepiali* (*P. hepiali*) is an important strain isolated from natural Chinese cordyceps [5,6]. Its mycelium can be prepared on a large scale through artificial submerged fermentation, and its core active components, such as polysaccharides, adenosine and mannitol, are highly similar to those of natural Chinese cordyceps [6,7,8], which has been approved for use in health food and related research. At present, many related derivatives have appeared on the market, such as Jinshuibao Capsules and Ningxinbao Capsules [9,10]. In addition to inheriting the traditional effects of Chinese cordyceps in tonifying the lung and kidney, they also exhibit significant effects such as enhancing body immunity, anti-fatigue effects [11], antioxidant effects [12], protecting liver and kidney functions [13], regulating blood lipids and assisting in anti-tumor effects [14]. Among these, the antioxidant effect, as one of their core pharmacological activities, can eliminate free radicals in the body, inhibit oxidative stress responses [15,16], and reduce the harm caused by oxidative damage to the body [17], and thus has important research significance in delaying aging and preventing chronic diseases [18,19].

Oxidative stress is the result of excessive reactive oxygen species (ROS) produced during the metabolic processes of the body [20], which can cause damage to cells [21], thereby inducing inflammatory responses, accelerating body aging [22], and even triggering various chronic diseases [23,24]. It is an important inducement leading to the decline of body functions and the occurrence of diseases. As one of the core indicators of the activity of medicinal fungi, antioxidant capacity is directly related to the level of their medicinal value [25,26,27], and it is also an important basis for evaluating their protective effect on the body [28]. Studies have shown that low-temperature stress can significantly affect the antioxidant capacity and secondary metabolite biosynthesis of medicinal fungi. For example, *Cordyceps militaris* exhibited reduced cordycepin content and altered lipid and organic acid metabolism pathways at 15 °C [29], while Volvariella volvacea showed dynamic changes in mannitol content and differential expression of genes related to trehalose metabolism under 0 °C conditions [30]. As a fungus isolated from natural Chinese cordyceps, the fermented products of *P. hepiali* are rich in active components and have potential antioxidant activity [31]. Therefore, clarifying the antioxidant capacity of *P. hepiali*‘s fermented products is of great significance for elucidating the effect of low-temperature stress on its active components and for promoting the medicinal development of this strain.

Mass spectrometry-based untargeted metabolomics is widely adopted to screen differential metabolites and reveal the biosynthetic mechanisms of active ingredients in medicinal fungi [32,33,34]. In most relevant studies, liquid chromatography is commonly used as the separation technique prior to mass spectrometric identification of metabolites. Existing research has shown that UHPLC-Q Exactive HF-X-based untargeted metabolomics distinguished the metabolite landscapes of *Cordyceps sinensis* from other *Cordyceps* species [32]. Relevant studies on *Ganoderma* species have also successfully identified various terpenoid compounds and clarified their structural characteristics and bioactivities using this approach [34,35], and multiomics revealed that temperature changes significantly affected flavonoid biosynthesis and the fermentation quality of *Ophiocordyceps sinensis* [36]. Despite these advances, untargeted metabolomics has rarely been applied to investigate the effects of different temperature treatments on the metabolic profiles of *P. hepiali*, and research on its temperature-responsive secondary metabolism remains insufficient. Our earlier work only conducted interspecific metabolic comparison of medicinal fungi without involving temperature stress treatments [37]. Combined with databases such as the Kyoto Encyclopedia of Genes and Genomes (KEGG) to carry out functional annotation and pathway enrichment analysis [38,39], this approach can clarify the core biological processes involved in differential metabolites, providing scientific data support for elucidating medicinal fungi activity mechanisms under varying temperature conditions.

In contrast, targeted metabolomics enables precise quantification of specific metabolites and compensates for the limitations of untargeted metabolomics in quantitative verification. Temperature significantly influences flavonoid accumulation in fungi; for example, cold storage increased flavonoids and phenol in Lentinula edodes via antioxidant accumulation against oxidative damage [40]. Similarly, low-temperature mycorrhizal inoculation boosts host phenol and flavonoids by elevating PAL activity [41]. However, the regulatory effects of different temperature treatments on flavonoid and phenolic compound biosynthesis in *P. hepiali* remain poorly understood. Targeted analysis of specific metabolites such as polysaccharides [42], adenosine and polyphenols [43,44] provides reliable quantitative data for verifying metabolic mechanisms. To elucidate the antioxidant capacity of *P. hepiali* fermented products, this study conducted in vitro antioxidant activity assays to systematically evaluate free radical scavenging efficiency and oxidative damage inhibition. Combined with flavonoid secondary metabolite mining, the antioxidant activity level was clarified, providing reliable experimental evidence for subsequent research.

Due to the lack of systematic research on temperature-regulated secondary metabolism in *P. hepiali*, this study combined untargeted and targeted metabolomics to analyze the changes in secondary metabolites, mainly flavonoids, in *P. hepiali* under different temperature treatments. Kyoto Encyclopedia of Genes and Genomes (KEGG) pathway enrichment analysis was used to screen key metabolic pathways related to temperature-regulated flavonoid biosynthesis. In addition, in vitro antioxidant tests were conducted to investigate the effects of temperature-induced flavonoid variation on the antioxidant performance of fermented *P. hepiali*. This work systematically describes the responses of the flavonoid metabolism and antioxidant activity of *P. hepiali* to temperature changes, aiming to provide experimental support for the optimized fermentation and metabolic regulation of this medicinal fungus.

## 2. Materials and Methods

### 2.1. Fungal Sample Materials

The original *P. hepiali* strain used in this study was isolated from Chinese cordyceps collected in Henan Mongolian Autonomous County, Qinghai Province (34°25′34″ N, 101°29′22″ E; altitude 3714), and was provided by the Academy of Animal Science and Veterinary Medicine, Qinghai University (Xining, China). The isolated *P. hepiali* strains were purified by solid culture and then subjected to shake-flask fermentation. On a sterile operating table, activated strains were carefully picked and transferred to sterilized Petri dishes, and then excess medium was removed. Twenty-five mycelial blocks with a length of approximately 0.2 cm were inoculated into conical flasks containing 500 mL of seed liquid (300 mL per flask), and placed in ZQLY-300N shaking incubators (Shanghai Zhichu Instrument Co., Ltd. Shanghai, China) for 7 days of shaking culture at 135 rpm under conditions of 17 °C, 20 °C, 23 °C, 26 °C, and 29 °C, respectively. Subsequently, they were transferred to newly prepared liquid medium and continuously fermented for 7 days under the same conditions. The seed liquid medium was prepared with 300 g potato, 30 g glucose, 4.5 g peptone, 3 g KH_2_PO_4_, and 0.3 g MgSO_4_, and cultivated in 300 mL/500 mL Erlenmeyer flasks. The liquid fermentation medium was prepared with 2100 g potato, 210 g glucose, 31.5 g peptone, 21 g KH_2_PO_4_, and 2.1 g MgSO_4_, also loaded into 300 mL/500 mL Erlenmeyer flasks for culture. After liquid fermentation was completed, samples were collected in 50 mL centrifuge tubes and centrifuged at 7000 rpm for 5 min at 18 °C using an H3-16RI centrifuge (Hunan Hexi Instrument Equipment Co., Ltd. Hunan, China). The supernatant was discarded, and the precipitate was stored at —80 °C in a DW-86W100 ultra-low-temperature freezer (Qingdao Haier Special Electric Co., Ltd. Qingdao, China) for subsequent analysis.

### 2.2. Determination of In Vitro Antioxidant Capacity and Bioactive Substances

Adenosine and mannitol reference standards were purchased from Shanghai Yuanye Bio-Technology Co., Ltd. (Shanghai, China) [45], and all analytical kits were obtained from Suzhou Comin Biotechnology Co., Ltd. (Suzhou, China). All physiological indices, including antioxidant enzyme activity, antioxidant substance content and radical scavenging capacity, were determined using commercial kits (Suzhou Comin Biotechnology Co., Ltd. Suzhou, China) following standard protocols [46]. Briefly, samples were homogenized in corresponding extraction buffers on ice, followed by centrifugation to obtain supernatants for subsequent colorimetric reaction and spectrophotometric detection at specific wavelengths. All calculations were performed according to the standard formulas provided by the kits with high correlation coefficients (Table S1). Adenosine and mannitol contents were quantified by HPLC using an Agilent 1260 system (Agilent Technologies, Santa Clara, CA, USA). Adenosine was extracted with 90% methanol and detected by VWD, while mannitol was extracted with ultrapure water and measured by RID. After ultrasonic extraction, centrifugation and membrane filtration, the contents were calculated based on external standard calibration curves.

### 2.3. Untargeted Metabolomics Analysis

#### 2.3.1. Sample Preparation and Extraction

Mycelia of *P. hepiali* underwent cryodesiccation treatment over a 63 h period via a Scientz-100F vacuum freeze-dryer (Ningbo, China). The desiccated material was crushed into consistent powder utilizing an MM 400 mixer mill at 30 Hz for 1.5 min (Retsch, Haan, Germany). Precisely 30 mg fungal powder was quantified via an MS105DM electronic analytical balance (Zurich, Switzerland). The extraction solution was pre-cooled 70% aqueous methanol (CAS: 67-56-1, chromatographic grade, Merck, Shanghai, China) at −20 °C with internal standard added. Steps to formulate internal standard working liquid are listed: 1 mg internal standard reference material was mixed into 1 mL 70% aqueous methanol to create a 1000 μg/mL stock liquid, then diluted using 70% methanol solution until the working concentration reached 250 μg/mL. Throughout the extraction procedure, vortex agitation was carried out at 30 min intervals, with each mixing session lasting 30 s, and this cycle was repeated six times in total. Once the extraction process concluded, the blended suspension underwent centrifugation at 12,000 rpm over a 3 min period. The resultant supernatant was subjected to filtration via a 0.22 μm micropore membrane, and the acquired filtrate was loaded into sample vials to conduct subsequent UPLC-MS/MS detection.

#### 2.3.2. UPLC-MS/MS Analysis

All test specimens underwent separation via an LC-30A ultra-high-performance liquid chromatograph (Shimadzu, Japan) installed with a Waters ACQUITY UPLC HSS T3 column (1.8 μm, 2.1 mm × 100 mm). The chromatographic column was maintained at a constant temperature of 40 °C, the mobile phase flow velocity was adjusted to 0.40 mL/min, and each injection dosage was fixed at 4 μL. Mobile phase A consisted of ultrapure water supplemented with 0.1% formic acid, while mobile phase B was composed of acetonitrile with an identical 0.1% formic acid additive. The prepared specimens were held within the autosampler at 4 °C during the whole detection process. To remove deviations caused by fluctuating instrument signals, specimens were measured one after another in random arrangements. Gradient elution parameters were set as listed below: 0–4 min, 95% A and 5% B; 5 min, 35% A and 65% B; 6–7.5 min, 1% A and 99% B; 7.6–10 min, 95% A and 5% B. Formic acid (CAS: 64-18-6, chromatographic grade) was supplied by Aladdin (Shanghai, China), and acetonitrile (CAS: 75-05-8, chromatographic grade) was purchased from Shanghai Xingke High-Purity Solvent Co., Ltd. (Shanghai, China). The chromatographic column remained stabilized at 40 °C, the mobile phase flow velocity was maintained at 0.40 mL/min, and each single injection quantity was adjusted to 3 μL.

Mass spectrometric detection was performed using an AB Triple TOF 6600 mass spectrometer (Shanghai Applied Protein Technology Co., Ltd., Shanghai, China). Data were acquired simultaneously in positive and negative ion modes with an acquisition time of 10 min. The ion source adopted two ionization potentials of 5000 V and −4000 V for positive and negative modes separately, with the source heating temperature fixed at 550 °C. The spray gas (Gas 1), auxiliary heating gas (Gas 2), and curtain gas were adjusted to pressures of 50 psi, 60 psi and 35 psi individually. The declustering potential was 80 V for ESI+ and −80 V for ESI−. The collision energy step was 15 V, with the MS1 collision energy set at 10 V for ESI+ and −10 V for ESI−. The mass scan range was 50–1250 Da for the first-order mass spectrometry (MS1) and 25–1250 Da for the second-order mass spectrometry (MS2).

### 2.4. Targeted Metabolomics Analysis

#### 2.4.1. Test Materials and Sample Pretreatment

The samples were first freeze-dried in a vacuum freeze-dryer at −50 °C under a vacuum degree ≤ 10 Pa for 24 h to remove water and ensure the extraction efficiency of subsequent metabolites. After drying, the samples were taken out and cooled to room temperature in a desiccator for later use. An appropriate amount of dried mycelial sample was placed into the grinding jar of an MM400 mixer mill (Retsch, Haan, Germany). Grinding was performed at 30 Hz for 1.5 min until the sample became a fine and homogeneous powder without obvious particles, which was reserved for subsequent use. Exactly 20 mg of the above powder sample was weighed using an AS 60/220.R2 electronic analytical balance (RADWAG, Radom, Poland) and transferred into a 1.5 mL sterile centrifuge tube. Subsequently, 500 μL of 70% methanol solution containing internal standard (final internal standard concentration: 0.4 mg/L) was accurately added, and the mixture was thoroughly vortex-mixed for 30 s using a MIX-200 vortex shaker (Shanghai, China) to fully immerse the powder sample in the extractant. The centrifuge tubes were placed in a KQ5200E ultrasonic cleaner (Jiangsu, China) for ultrasonic extraction at room temperature with an ultrasonic power of 200 W for 30 min. During extraction, the tubes were gently inverted and mixed once every 10 min to ensure sufficient dissolution of metabolites. After sonication, the samples were centrifuged in a 5424R centrifuge (Hamburg, Germany) at 12,000 rpm and 4 °C for 5 min. Following centrifugation, the clarified supernatant was gently drawn with a pipette to prevent disturbance of the precipitate at the tube bottom. The supernatant was slowly dropped onto a 0.22 μm organic-phase filter membrane for filtration. The filtered sample was collected into a clean sample vial, sealed, and stored at 4 °C in a refrigerator for subsequent LC-MS/MS analysis.

A series of standard solutions of target metabolites with different concentrations (0.0005 μmol/L, 0.001 μmol/L, 0.005μmol/L, 0.01 μmol/L, 0.02 μmol/L, 0.05 μmol/L, 0.1 μmol/L, 0.2 μmol/L, 0.5 μmol/L, 1 μmol/L, 2 μmol/L, 5 μmol/L, 10 μmol/L, 20 μmol/L) were prepared and analyzed by injection under optimized LC-MS/MS conditions to obtain quantitative ion chromatographic peak intensity data corresponding to each standard concentration. Standard curves for each target compound were plotted and linear regression equations were established using the concentration ratio of external standard to internal standard (or external standard concentration) as the abscissa and the peak-area ratio of external standard to internal standard (or external standard peak area) as the ordinate. The results showed that all detected flavonoids and their derivatives exhibited good linear relationships within the tested concentration range, with linear regression equations and correlation coefficients shown in Table S2.

#### 2.4.2. LC-MS/MS Analysis

Data acquisition was carried out on a SCIEX ExionLC™ AD ultra-high-performance liquid chromatograph (SCIEX, Framingham, MA, USA) interfaced with a QTRAP^®^ 6500+ tandem mass spectrometry system (SCIEX, Framingham, MA, USA). Chromatographic separation was carried out on a Waters ACQUITY UPLC HSS T3 C18 column (1.8 μm, 100 mm × 2.1 mm i.d.; Waters Corporation, Milford, MA, USA). Ultrapure water supplemented with 0.05% formic acid served as mobile phase A, whilst acetonitrile with 0.05% formic acid acted as mobile phase B. The mobile phase flow velocity was set to 0.35 mL/min, the column temperature was maintained at 40 °C, and the injection dosage was 2 μL. Gradient elution was configured according to the following schedule: 0 min, A/B = 90:10 (*v*/*v*); 1 min, A/B = 80:20 (*v*/*v*); 9 min, A/B = 30:70 (*v*/*v*); 12.5 min, A/B = 5:95 (*v*/*v*); 13.5 min, A/B = 5:95 (*v*/*v*); 13.6 min, A/B = 90:10 (*v*/*v*); 16 min, A/B = 90:10 (*v*/*v*). Mass spectrum analysis was conducted using an electrospray ionization source held at 550 °C. The spray voltage was 5500 V in positive ion mode and −4500 V in negative ion mode, with the curtain gas pressure maintained at 35 psi. Each target ion pair was scanned on the QTRAP^®^ 6500+ system using optimized declustering potential and collision energy parameters. Qualitative analysis of mass spectrometry data was performed by constructing the MWDB (Metware Database) based on authentic standards. Quantitative determination was implemented via the multiple reaction monitoring (MRM) mode of triple-quadrupole mass spectrometers. Characteristic fragment ions were generated by induced ionization after screening target precursor ions to eliminate interfering factors and improve quantitative accuracy and repeatability. Mass spectral datasets were processed by the Analyst 1.6.3 and MultiQuant 3.0.3 software packages. Chromatographic peak integration was corrected according to the retention time and peak shape characteristics of standards, and quantification of target metabolites was completed with standard curves to ensure the accuracy and reliability of qualitative and quantitative results.

### 2.5. Data Preprocessing and Statistical Analysis

All quantitative results of the antioxidant activity assays conformed to a normal distribution, and Pearson correlation analysis was subsequently performed on the mean values of all physiological and biochemical indicators to explore their interrelationships. Data compilation and preliminary categorization were conducted in Microsoft Excel 2024. One-way analysis of variance (ANOVA) and significance difference analysis were subsequently performed via IBM SPSS Statistics 26.0, with the statistical significance threshold set at *p* < 0.05. ProteoWizard was utilized to convert raw MS data to mzML, and peak extraction and alignment as well as retention time calibration were accomplished via the XCMS platform. Features with >50% missing values were removed. Missing values were imputed by minimum/5 for >50% blank loss and KNN otherwise; peak areas were calibrated using SVR. Metabolite annotation was matched via in-house databases, public repositories, and metDNA. Only compounds with an identification score ≥ 0.5 and a QC coefficient of variation (CV) < 0.5 were retained for subsequent analysis. Data from positive/negative ion modes were merged, keeping top-ranked, low-CV metabolites for downstream analysis. Significance tests were conducted using SPSS 26.0. Multivariate statistical analyses including principal component analysis (PCA) and orthogonal partial least-squares discriminant analysis (OPLS-DA) were performed in R 4.2.0 to screen differential metabolites between groups. In addition, Venn diagrams, volcano plots and HCA clustering heatmaps were generated and analyzed via the cloud platform (https://cloud.metware.cn/, accessed 15 March 2026) for visual analysis. Using the KEGG database (https://www.genome.jp/kegg/, retrieved 25 March 2026), functional annotation and metabolic pathway enrichment analysis were performed on the screened differential metabolites to elucidate the biological metabolic pathways and regulatory mechanisms involved. Targeted metabolites were quantified by triple-quadrupole MS under SRM. Peak processing and quantification with standard curves were performed using Analyst 1.6.3 and MultiQuant 3.0.3, with contents calculated from standard regression formulas. Correlation analysis was performed using Chiplot software v2.6.1 (https://www.chiplot.online/, accessed 16 April 2026).

## 3. Results

### 3.1. Effects of Temperature on Antioxidant Capacity and Active Components of P. hepiali

**Figure 1:**
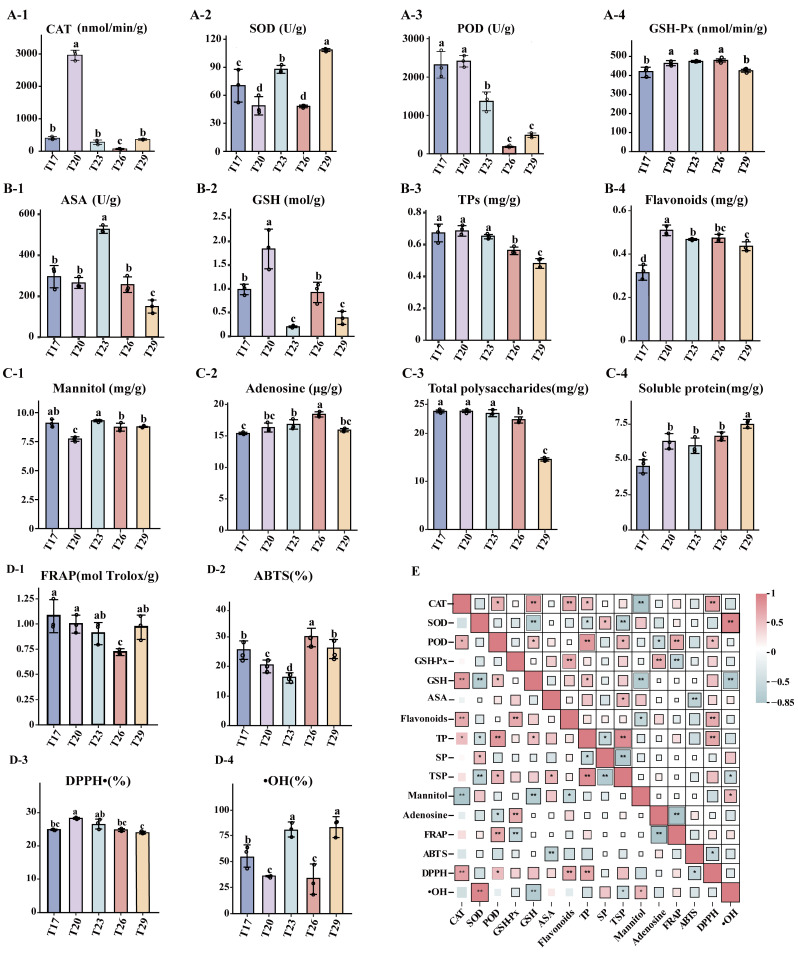
Antioxidant capacity of *P. hepiali* under different temperatures; (**A-1**) catalase (CAT); (**A-2**) superoxide dismutase (SOD); (**A-3**) peroxidase (POD); (**A-4**) glutathione peroxidase (GSH-Px); (**B-1**) ascorbic acid (AsA); (**B-2**) reduced glutathione (GSH); (**B-3**) total phenolic content (TPs); (**B-4**) flavonoid content; (**C-1**) mannitol content; (**C-2**) adenosine content; (**C-3**) total polysaccharides content (TSP); (**C-4**) soluble protein content (SP); (**D-1**) ferric reducing antioxidant power (FRAP); (**D-2**) ABTS radical scavenging activity; (**D-3**) 2,2-diphenyl-1-picrylhydrazyl radical scavenging capacity (DPPH•); (**D-4**) hydroxyl radical scavenging capacity (•OH); vertical bars represent mean ± standard deviation (SD). Columns marked with different letters indicate statistically significant differences (*p* < 0.05). (**E**) Pearson correlation analysis of all measured indicators. The analysis was performed using the mean values of each parameter under different temperature treatments. An asterisk “*” means significant correlation at *p* < 0.05, and a double asterisk “**” represents extremely significant correlation at *p* < 0.01.

**Figure 2:**
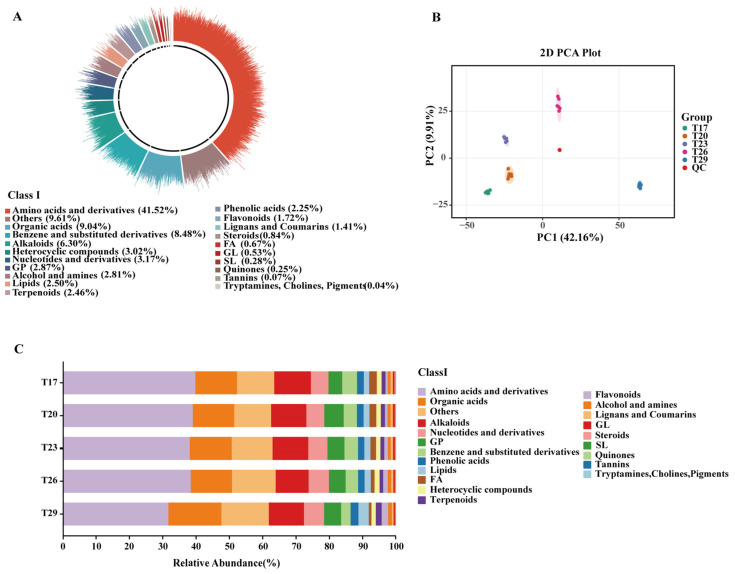
Metabolite distribution characteristics and multivariate statistical analysis of *P. hepiali*. (**A**) Proportion distribution of metabolite superclasses; (**B**) PCA plot of T17, T20, T23, T26, T29 and QC samples; (**C**) Relative abundance of superclass metabolites in each group.

**Figure 3:**
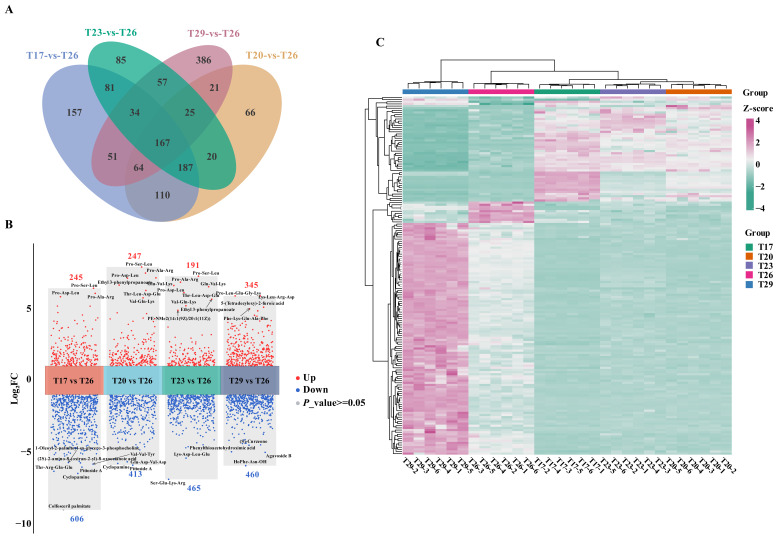
Analysis of key DAMs in *P. hepiali*. (**A**) Venn diagram of T17 vs. T26, T20 vs. T26, T23 vs. T26 and T29 vs. T26. (**B**) Volcano scatter plots of four comparison groups. Each panel corresponds to one comparison; the y-axis represents log_2_FC. Red points represent significantly up-regulated metabolites; blue points represent significantly down-regulated metabolites; gray points represent non-significant metabolites (*p* ≥ 0.05). (**C**) Clustering heatmap of major DAMs in each group.

**Figure 4:**
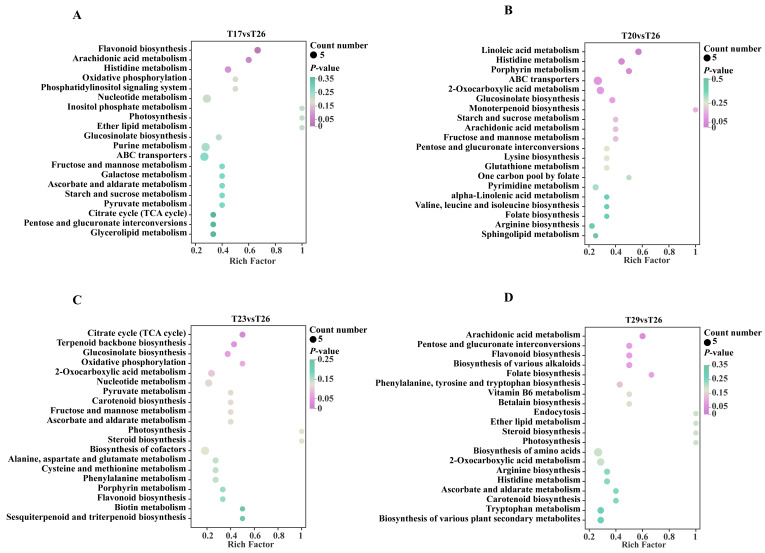
Top 20 differentially enriched metabolic pathways of *P. hepiali* in four comparison groups. (**A**) Top 20 pathways of T17 vs. T26; (**B**) top 20 pathways of T20 vs. T26; (**C**) top 20 pathways of T23 vs. T26; (**D**) top 20 pathways of T29 vs. T26.

The antioxidant performance of *P. hepiali* under various temperatures was evaluated using a set of indicators, including antioxidant enzymes, non-enzymatic antioxidants and total antioxidant capacity. Based on the quantification of 16 antioxidant-related indicators (Figure 1(A-1–D-4)), catalase (CAT) activity of *P. hepiali* peaked at T20 (Figure 1(A-1)), with a value of 2961.73 ± 155.14 nmol/min/g, approximately 45-fold that of the control group T26. For superoxide dismutase (SOD), the maximum activity of 108.53 ± 1.51 U/g was detected at T29, which was markedly higher than the T26 control (48.19 ± 1.41 U/g). T17, T20 and T23 yielded significantly higher peroxidase (POD) activity compared with T26 and T29 (Figure 1(A-3)). Glutathione peroxidase (GSH-Px) activity varied moderately across all temperature groups and rose progressively to reach its maximum at T26, before declining to 424.91 ± 7.38 nmol/min/g under the T29 treatment (Figure 1(A-4)). In terms of non-enzymatic antioxidants, ascorbic acid (ASA) content was the highest at T23 (524.59 ± 18.12 U/g), significantly exceeding the T26 level; by contrast, T29 produced the minimum ASA content of 146.81 ± 32.05 U/g, which differed statistically from all other groups (*p* < 0.05, Figure 1(B-1)). Reduced glutathione (GSH) accumulated to the highest level at T20 with obvious statistical differences against remaining treatments (*p* < 0.05), while GSH contents dropped sharply to 0.20 ± 0.02 μmol/g (T23) and 0.39 ± 0.14 μmol/g (T29), respectively (Figure 1(B-2)).

Total phenols (TPs) and flavonoid contents generally declined as cultivation temperature increased (Figure 1(B-3,B-4)). Intriguingly, T17 achieved the maximum TPs concentration (0.6727 ± 0.0642 mg/g) but the lowest flavonoid level (0.3803 ± 0.0168 mg/g). Temperature treatments also exerted prominent effects on the accumulation of key bioactive ingredients in *P. hepiali* mycelia (Figure 1(C-1–C-4)). Adenosine and mannitol contents showed limited fluctuations among tested groups (Figure 1(C-1,C-2)). Total polysaccharide (TSP) contents remained high and comparable at T17, T20, T23 and T26 (24.58 ± 0.29 mg/g, 24.61 ± 0.28 mg/g, 24.15 ± 0.72 mg/g and 22.86 ± 0.74 mg/g, respectively), followed by a sharp reduction to 14.57 ± 0.34 mg/g at T29 (Figure 1(C-3)). Soluble protein (SP) content increased gradually with rising temperature and peaked at T29 (7.55 ± 0.27 mg/g), a value 34.1% higher than the control, with a significant statistical difference (*p* < 0.05, Figure 1(C-4)). Regarding antioxidant capacity and free radical scavenging performance, the ferric reducing antioxidant power (FRAP) of T17, T20, T23 and T29 was remarkably superior to T26, with significant intergroup differences (*p* < 0.05, Figure 1(D-1)). The T26 group possessed the highest 2,2′-Azino-bis (3-ethylbenzothiazoline-6-sulfonic acid) radical scavenging rate (ABTS) (over 30%), significantly outperforming other treatments, whereas T23 displayed the weakest ABTS scavenging capacity (Figure 1(D-2)). The 2,2-Diphenyl-1-picrylhydrazyl radical scavenging rate (DPPH•) declined overall with elevated temperature (Figure 1(D-3)), with T20 registering the peak value of 28.30%, marginally above T26 (24.84%). No significant disparities in DPPH• scavenging were observed among T17, T23 and T29 (*p* > 0.05). The hydroxyl radical (•OH) scavenging rates of T17, T23 and T29 all exceeded 50%; notably, T23 and T26 attained scavenging efficiencies above 80%, markedly higher than other experimental groups (*p* < 0.05, Figure 1(D-4)).

Subsequently, correlation analysis was conducted on all measured antioxidant indices (Figure 1E). The results revealed that both TPs and flavonoid contents were significantly positively correlated with DPPH•, GSH and CAT levels, yet negatively correlated with ABTS scavenging activity. Collectively, we infer that the antioxidant potential of *P. hepiali* under varied thermal conditions is tightly associated with its endogenous metabolites, especially phenolic and flavonoid compounds.

### 3.2. Metabolomic Analysis of P. hepiali Under Different Temperatures

Untargeted metabolomics detection was performed to obtain total ion current chromatograms (TICs) of *P. hepiali* across five temperature gradients. Overlapped TIC profiles of quality control samples exhibited consistent retention time and peak intensity (Figure S1A,B), verifying good repeatability and reliability of the UPLC-MS/MS analytical method. Metabolic profiling results (Figure 2A) showed that a total of 2842 metabolites were identified and annotated, which were classified into 21 superclasses and 78 classes (Table S3). The most abundant superclass was amino acids and their derivatives (1180 metabolites, accounting for 41.52%), followed by organic acids (257 metabolites, 9.04%), benzene and its derivatives (241 metabolites, 8.48%), alkaloids (179 metabolites, 6.3%), heterocyclic compounds (91 metabolites, 3.02%), nucleotides and derivatives (90 metabolites, 3.17%) and terpenoids (70 metabolites, 2.46%). Notably, phenolic metabolites including flavonoids (49 metabolites, 1.72%), phenolic acids (64 metabolites, 2.25%), lignans and coumarins (40 metabolites, 1.41%) collectively accounted for 5.38%. Analysis of the relative abundance of Class I metabolites showed that *P. hepiali* exhibited a distinct metabolite accumulation pattern under five temperature treatments. It maintained high metabolic stability from T17 to T26, whereas the metabolite accumulation under T29 treatment changed significantly compared with the other four groups. The metabolic profile at 26 °C was dominated by amino acids and their derivatives. Temperatures of 17–23 °C primarily induced the accumulation of phenolic acids and alkaloids and inhibited flavonoid biosynthesis. At 29 °C, significant depletion of amino acids and massive accumulation of organic acids occurred, along with the synergistic accumulation of flavonoids and phenolic acids (Figure 2C). To further investigate metabolic differences in *P. hepiali* under different temperature treatments, principal component analysis (PCA) was performed. The results showed (Figure 2B) that PC1 and PC2 were 42.16% and 9.91%, respectively. Each temperature group exhibited obvious separation without intergroup overlap. T29 deviated remarkably from other groups. QC samples clustered closely, suggesting good repeatability and reliability of data acquisition. Compared with PCA, OPLS-DA eliminates irrelevant variations to screen differential variables and possesses superior classification and prediction capacity. In comparison groups of T17 vs. T26, T20 vs. T26, T23 vs. T26 and T29 vs. T26, the experimental groups and control group achieved thorough separation along the T score axis, and samples within each group clustered tightly (Figure S2A). The T score of T29 vs. T26 had the highest explanatory value of 50.3% (Figure S2(A3)). After 200 permutations, Q^2^ = 0.989, R^2^Y = 1, R^2^X = 0.56 in the T17 vs. T26 comparison group (Figure S2(B1)); Q^2^ = 0.979, R^2^Y = 1, R^2^X = 0.496 in T20 vs. T26 (Figure S2(B2)); Q^2^ = 0.981, R^2^Y = 1, R^2^X = 0.506 in T23 vs. T26 (Figure S2(B3)); and Q^2^ = 0.988, R^2^Y = 1, R^2^X = 0.561 in T29 vs. T26 (Figure S2(B4)). All permutation Q^2^ values were negative, and the original model’s Q^2^ exceeded permutation outputs, excluding overfitting. R^2^Y = 1 denoted excellent model interpretability, validating significant intergroup metabolic divergence.

### 3.3. Screening of Differential Accumulated Metabolites (DAMs) and Hierarchical Cluster Analysis (HCA)

Differential accumulated metabolites (DAMs) were screened based on VIP ≥ 1 and *p* < 0.05. A Venn diagram, volcano plot and clustering heatmap were adopted to visually analyze the similarity of and differences in metabolites among four sample groups (Figure 3A–C). The Venn diagram showed (Figure 3A) that 167 DAMs were commonly differentially expressed in all temperature comparison groups, forming the core regulatory network. These metabolites covered 17 categories, including amino acids and their derivatives, organic acids, terpenoids, phenolic acids, flavonoids, and lipids. Notably, T29 vs. T26 possessed the largest number of 386 DAMs, followed by 157 DAMs in T17 vs. T26 and 110 DAMs in T23 vs. T26, and T20 vs. T26 had the minimum 66 DAMs. To further analyze the expression patterns and statistical significance of DAMs in each temperature comparison group (Figure 3B), systematic clustering of all DAMs was performed based on VIP Top50 (Figure S3). The results showed that there were 851 DAMs in T17 vs. T26 (245 up-regulated, 606 down-regulated), 678 DAMs in T20 vs. T26 (247 up-regulated, 431 down-regulated), 656 DAMs in T23 vs. T26 (191 up-regulated, 465 down-regulated), and 805 DAMs in T29 vs. T26 (345 up-regulated, 460 down-regulated). The significantly up- and down-regulated metabolites included amino acids and their derivatives, organic acids, terpenoids (such as some alkaloids), phenolic acids, flavonoids, and lipids. Flavonoid metabolites were mainly up-regulated in T17 and T20, while they were down-regulated in T29. Among the phenolic acids, 2,4-dihydroxybenzoic acid was specifically up-regulated in T17. Unsupervised hierarchical clustering analysis was conducted based on DAMs of four comparison groups to systematically clarify the overall expression and sample clustering relationship of metabolites at different temperatures (Figure 3C). Amino acid metabolites accounted for more than 50%. Flavonoids including Texasin, Artemetin and Chrysin were up-regulated in T17 and T20, and down-regulated in T29. 2,4-dihydroxybenzoic acid was enriched in T17 while caffeic acid accumulated largely in T29. Acylcarnitines and nucleotide derivatives were highly expressed in T23 and T29, and phospholipids showed the most dramatic changes in T23, presenting temperature-dependent changes.

### 3.4. KEGG Annotation and Metabolite Enrichment Analysis

**Figure 5:**
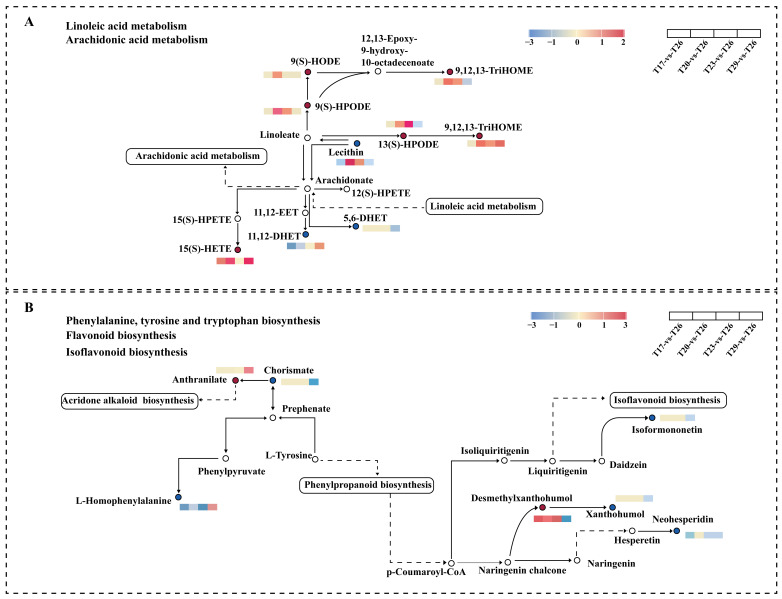
KEGG pathways of key metabolites in four groups of *P. hepiali* samples. Colored squares before each metabolite represent corresponding log_2_FC values. (**A**) KEGG map of linoleic acid metabolism and arachidonic acid metabolism; (**B**) KEGG map of flavonoid biosynthesis and its upstream and downstream pathways. Note: Red dots indicate significantly up-regulated metabolites, blue dots indicate significantly down-regulated metabolites, and blank circles represent undetected metabolites.

The KEGG database was used to analyze gene functions and metabolic pathways. Combined with the top 20 enriched metabolic pathways of DAMs in each group, the results showed that DAMs of T17 vs. T26 and T29 vs. T26 involved 70 pathways each, while those of T20 vs. T26 and T23 vs. T26 involved 61 pathways each. The main pathways are displayed in bubble plots (Figure 4A–D). In addition, we noticed that the metabolic pathway of flavonoid biosynthesis existed in both the T17 vs. T26 and T29 vs. T26 comparison groups (Figure 4A,D). The metabolic pathways of linoleic acid metabolism and arachidonic acid metabolism were significantly enriched in T17 vs. T26, T20 vs. T26 and T29 vs. T26 (Figure 4A,B,D). The TCA cycle pathway was significantly enriched in the T23 vs. T26 comparison group (Figure 4C). Furthermore, the most relevant overlapping metabolic pathways of DAMs were screened based on KEGG and enrichment maps to summarize metabolic regulation changes (Figure 5). In linoleic acid metabolism and arachidonic acid metabolism pathways, 15(S)-HETE, 9(S)-HODE, 9,12,13-TriHOME, 9(S)-HPODE and 13(S)-HPODE were markedly up-regulated, while lecithin, 11,12-DHET and 5,6-DHET were obviously down-regulated (Figure 5A). In the flavonoid biosynthesis pathway (Figure 5B), desmethylxanthohumol was significantly up-regulated, while xanthohumol and neohesperidin were markedly down-regulated. In the upstream phenylalanine, tyrosine and tryptophan biosynthesis pathway, anthranilate increased, whereas chorismate and L-homophenylalanine decreased. Isoformononetin was distinctly down-regulated in the downstream isoflavonoid biosynthesis pathway. In conclusion, temperature alterations dynamically regulate core metabolic pathways such as flavonoid biosynthesis, linoleic acid and arachidonic acid metabolism, as well as the TCA cycle, leading to differential metabolite accumulation.

### 3.5. Combined Analysis of Key Flavonoids and Antioxidant Components

**Figure 6:**
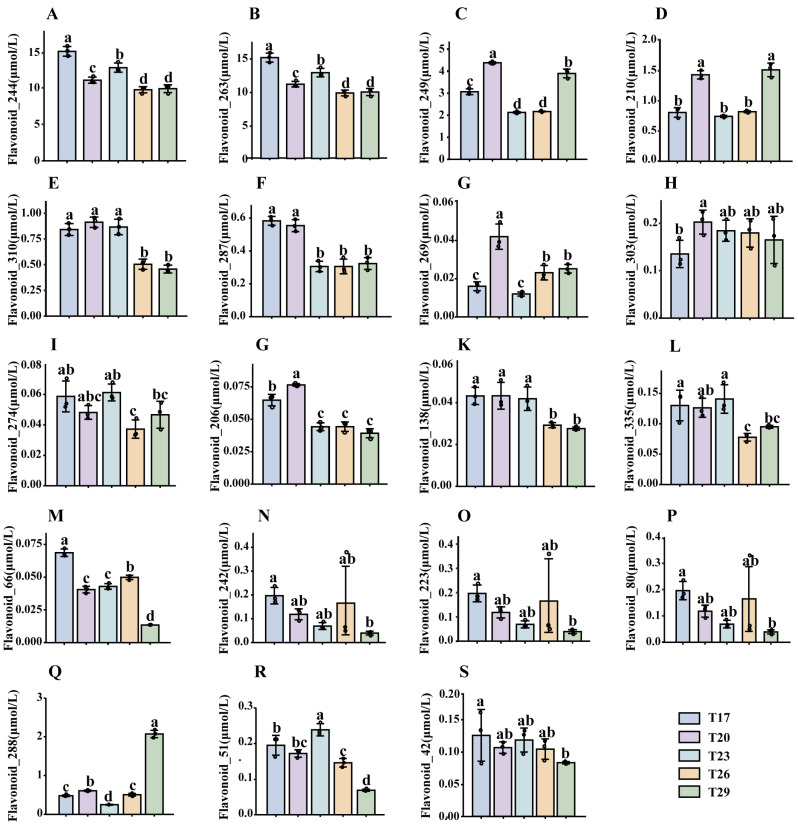
Relative contents of 19 differential flavonoid metabolites across five temperature groups. Error bars represent standard deviation, and different lowercase letters denote significant differences among groups (*p* < 0.05). (**A**) Chlorogenic acid (Flavonoid_244); (**B**) Cryptochlorogenic acid (Flavonoid_263); (**C**) Protocatechuic acid (Flavonoid_249); (**D**) Gentisic acid (Flavonoid_210); (**E**) Quercetin 3,7-diglucoside (Flavonoid_310); (**F**) Sinapinic acid (Flavonoid_287); (**G**) carlinoside (Flavonoid_269); (**H**) Dihydrokaempferol 3-O-glucoside (Flavonoid_303); (**I**) Camelliaside A (Flavonoid_274); (**J**) Salicylic acid (Flavonoid_206); (**K**) Baimaside (Flavonoid_138); (**L**) Vanillin (Flavonoid_335); (**M**) Puerarin (Flavonoid_66); (**N**) Gallocatechin gallate (Flavonoid_242); (**O**) Epigallocatechin gallate (Flavonoid_223); (**P**) (-)-Gallocatechin gallate (Flavonoid_80); (**Q**) Caffeic acid (Flavonoid_288); (**R**) 3′-Methoxypuerarin (Flavonoid_51); (**S**) Nicotiflorin (Flavonoid_42).

**Figure 7:**
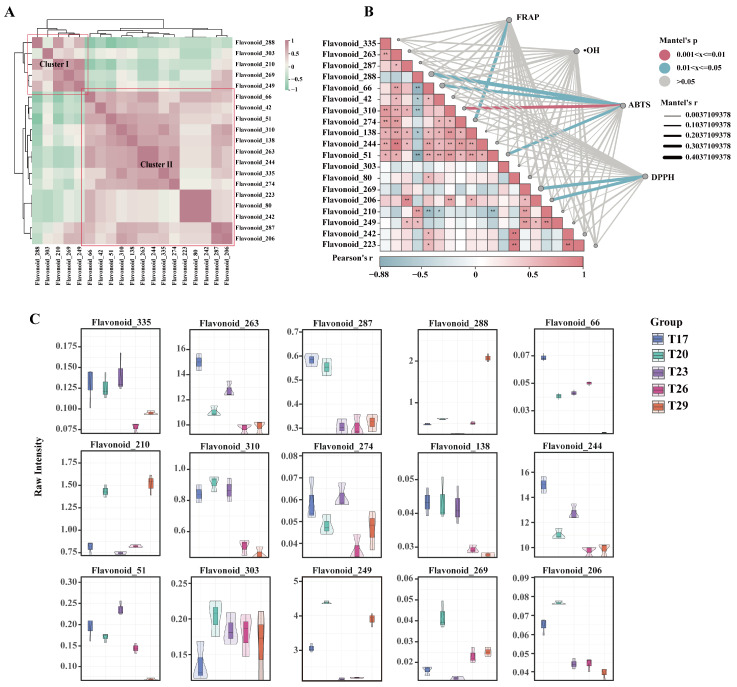
Analysis on accumulation patterns of flavonoid metabolites and their correlation with antioxidant activity of *P. hepiali* under different temperature treatments. (**A**) Hierarchical clustering heatmap illustrating temperature response profiles of metabolites. Major clusters are marked with red frames. (**B**) Correlation network between flavonoid metabolites and antioxidant indicators analyzed by Mantel test. Line colors represent significance levels (red: *p* < 0.001, blue: 0.001 < *p* < 0.05, gray: *p* > 0.05), and line thickness corresponds to Mantel’s r value. An asterisk “*” means significant correlation at *p* < 0.05, and a double asterisk “**” represents extremely significant correlation at *p* < 0.01. Pearson correlation coefficient matrix of metabolites is displayed at bottom left. (**C**) Box plots of key differential metabolites reflecting intrasample data distribution and median variation.

Based on antioxidant capacity detection and untargeted metabolomics analysis, significant differences in flavonoid contents were observed in *P. hepiali*. Targeted metabolomics of flavonoids and phenolic compounds was further performed to verify their effects on fungal antioxidant capacity. A total of 56 flavonoid and phenolic metabolites were identified and quantified in *P. hepiali* (Table S4). A *T*-test was applied to screen out 19 shared differential metabolites across different groups. Flavonoid metabolites exhibited distinct accumulation patterns under five temperature treatments of 17 °C, 20 °C, 23 °C, 26 °C and 29 °C (Figure 6A). Vanillin (Figure 6L), cryptochlorogenic acid (Figure 6B) and sinapinic acid (Figure 6F) accumulated abundantly at 17 °C and declined gradually with rising temperature. By contrast, 3′-methoxypuerarin (Figure 6R) and gallocatechin gallate(Figure 6N) reached their maximum levels at 23 °C and 26 °C, while most flavonoids decreased markedly at 29 °C. HCA further divided metabolites into two major clusters (Figure 7A). Cluster I, including caffeic acid, dihydrokaempferol 3-O-glucoside and gentisic acid, showed high abundance under low temperature and declined as temperature increased. Cluster II, such as epigallocatechin gallate, gallocatechin gallate and (-)-gallocatechin gallate, accumulated predominantly at 23 °C and 26 °C. The correlation network based on the Mantel test (Figure 7B) revealed that vanillin, cryptochlorogenic acid and sinapinic acid exhibited extremely significant positive correlations with FRAP, DPPH• and •OH (*p* < 0.001). Despite massive accumulation at higher temperatures, epigallocatechin gallate, gallocatechin gallate and other compounds showed weak correlations with all antioxidant indices (*p* > 0.05). Combined with accumulation characteristics and correlation analysis, flavonoids with pronounced temperature-dependent variations were selected for visualization analysis (Figure 7C). Vanillin presented the highest median level at 17 °C and decreased stepwise with temperature elevation. Sinapinic acid peaked sharply at 23 °C and dropped to the minimum at 29 °C. 3′-methoxypuerarin and gallocatechin gallate maintained relatively high contents at 26 °C and 29 °C. These results indicated that 26 °C serves as the balance point for flavonoid biosynthesis in *P. hepiali*. The fungus may activate specific flavonoid branches under high temperature to compensate for the loss of overall antioxidant capacity, whereas low temperature facilitates the accumulation of flavonoids with strong antioxidant activity.

## 4. Discussion

Temperature is a key factor governing *P. hepiali* fermentation via modulating mycelial metabolism and active substance synthesis [47]. By culturing strains at different temperatures combined with metabolomics, we uncovered temperature-mediated flavonoid regulation, and four antioxidant enzymes displayed distinct temperature responses [48,49,50]. Consistent with prior fungal stress studies, moderate low temperature (20 °C) improved CAT activity, and 17–23 °C elevated POD activity to relieve low-temperature lipid peroxidation [51,52]. In line with previous findings, high temperature (29 °C) maximized SOD activity to eliminate heat-induced superoxide anions and oxidative damage [53]. GSH-Px was insensitive to temperature changes and peaked at 26 °C, with stable activity depending on continuous GSH supply, as confirmed by their positive correlation in the glutathione antioxidant cycle [54]. Moreover, the negative correlations of SOD with CAT and POD, and the positive correlation between CAT and POD, reveal a synergistic and differentiated regulatory pattern of the fungal antioxidant enzyme system under temperature fluctuation [55,56,57]. High temperature induced massive ROS accumulation, which significantly consumed endogenous non-enzymatic antioxidants ASA and GSH and minimized their contents at 29 °C. Such heat-induced antioxidant depletion and synthesis inhibition have been widely reported in previous studies [58,59]. Additionally, temperature distinctly regulates the accumulation of secondary metabolites. Low temperature facilitated TPs synthesis by maintaining phenylpropane metabolic enzyme activity, while high temperature suppressed related enzymatic activity and reduced TPs content [27,60,61]. Flavonoids peaked at 20 °C and were positively correlated with DPPH•, CAT and GSH-Px. As critical radical scavengers, flavonoids enhance mycelial antioxidant capacity by regulating antioxidant enzyme activities under temperature stress [62]. A sharp decline in total polysaccharides at 29 °C, together with elevated SOD and lowered non-enzymatic antioxidants, suggests high temperature inhibits polysaccharide biosynthesis and boosts ROS accumulation [63,64]. Soluble protein rose with temperature and peaked at 29 °C, an adaptive strategy for cellular homeostasis under heat stress [63]. Differing antioxidant constituents lead to varied radical scavenging efficiency, and overall antioxidant capacity depends on synergistic interactions between these compounds [65]. In summary, temperature profoundly affects the antioxidant properties of fermentation products by regulating the synergistic expression of enzymatic and non-enzymatic antioxidant systems in *P. hepiali*.

Our untargeted metabolomics analysis revealed obvious metabolic discrepancies in *P. hepiali* under different temperature treatments, with predominant metabolites including amino acids, organic acids, nucleotides, phenols and flavonoids [8]. PCA separated samples by temperature, and the T29 vs. T26 comparison contained the most differential metabolites, demonstrating that high temperature imposes the strongest metabolic imbalance [66]. KEGG enrichment analysis indicated that temperature significantly affected flavonoid biosynthesis [50], with low temperature promoting flavonol and flavonoid glycoside formation, whereas high temperature remodeled isoflavonoid profiles [67,68]. Universal enrichment of linoleic and arachidonic acid metabolism, plus elevated lipid peroxidation markers and reduced lecithin, proved temperature-triggered ROS accumulation, lipid peroxidation and membrane injury [69]. Collectively, divergent pathway activation implies *P. hepiali* launches antioxidant defense upon membrane damage via phenylpropane rearrangement, altering mycelial physiology and fermented product quality [70]. It reveals complex adjustments of the phenylpropane metabolic network under temperature variation [70], further affecting the physiological state of mycelia and functional quality of fermentation products.

Targeted metabolomics further characterized temperature-dependent accumulation of phenolics and flavonoids and their antioxidant relevance in *P. hepiali* [36]. Hierarchical clustering grouped T17/T20 together and separated T29, revealing divergent flavonoid biosynthetic pathways under low and high temperature [67]. Matching untargeted metabolome trends, targeted quantification found vanillin, cryptochlorogenic acid and sinapinic acid accumulated at low temperature and strongly correlated with FRAP, DPPH• and •OH, dominating cold-triggered antioxidant improvement [71]. Sinapinic acid peaked at 23 °C rather than 17 °C; phenolic acids tolerated mild warming whereas vanillin preferred low temperature [72]. Most flavonoids decreased sharply at 29 °C, except 3′-Methoxypuerarin and gallocatechin gallate. Coupled with high SOD but depressed non-enzymatic antioxidants at 29 °C, the fungus selectively preserves partial flavonoid biosynthesis to maintain basal antioxidant defense under heat stress [73]. Overall, 26 °C serves as the dynamic equilibrium point for flavonoid synthesis in *P. hepiali*. Low temperature promotes the accumulation of high-antioxidant phenolic acids, whereas temperatures deviating from 26 °C reduce synthetic efficiency and reshape metabolite profiles. These temperature-dependent metabolic features provide a theoretical basis for optimizing the antioxidant quality of fermented *P. hepiali* products through precise temperature regulation.

## 5. Conclusions

This study metabolomically clarified the temperature-dependent regulation of flavonoid biosynthesis and antioxidant capacity in *P. hepiali*. Our results showed that temperature shifts reshaped flavonoid biosynthesis, fatty acid metabolism and the TCA cycle to balance antioxidant buildup and energy metabolism for tiered antioxidant defense. KEGG enrichment indicated that both low and high temperatures activated flavonoid biosynthesis, while temperature-induced ROS accumulation and membrane injury altered fatty acid metabolism. TCA cycle enrichment at 23 °C ensured sufficient energy supply. Targeted quantification further confirmed that temperature modulated phenolic and flavonoid accumulation, and key metabolites including vanillin, cryptochlorogenic acid and sinapinic acid were strongly positively correlated with antioxidant capacity. Overall, *P. hepiali* adopts hierarchical metabolic strategies to adapt to temperature changes. This work elucidates the thermal adaptation mechanism of medicinal fungi and provides a theoretical basis for improving the functional quality of fermented products via precise temperature control.

## Data Availability

Dataset available from the corresponding author upon request. The dataset is large and has not been uploaded temporarily.

## Supplementary Materials

The following supporting information can be downloaded at https://www.mdpi.com/article/10.3390/antiox15070892/s1: Figure S1: (**A**): Total ion chromatograms (TICs) of QC, 17 °C, 20 °C, 23 °C, 26 °C and 29 °C under positive mode; (**B**): total ion chromatograms (TICs) of QC, 17 °C, 20 °C, 23 °C, 26 °C and 29 °C under negative mode; Figure S2: Score plots and permutation test validation of OPLS-DA between different temperature treatment groups and the T26 group; Figure S3: HCA for the top 50 differential metabolites in the T17 vs T26, T20 vs T26, T23 vs T26 and T29 vs T26 comparison groups; Table S1: Detection parameters of active ingredient content and antioxidant activity of *P. hepiali*; Table S2: Standard curves and linear parameters for quantitative detection of flavonoid metabolites; Table S3: Summary of metabolite classification statistics in untargeted metabolomics analysis of *P. hepiali*; Table S4: Quantitative identification of 56 flavonoid and phenolic metabolites in *P. hepiali*; red-labeled compounds indicate 19 differential flavonoids common to four comparison groups.

## Abbreviations

The following abbreviations are used in this manuscript:

*P. hepiali*	*Paecilomyces hepiali*
KEGG	Kyoto Encyclopedia of Genes and Genomes
CAT	Catalase
POD	Peroxidase
SOD	Superoxide dismutase
GSH-Px	Glutathione peroxidase
ASA	Ascorbic acid
GSH	Reduced glutathione
TPs	Total phenols
TSP	Total polysaccharide
SP	Soluble protein
FRAP	Ferric reducing antioxidant power
ABTS	2,2′-Azino-bis(3-ethylbenzothiazoline-6-sulfonic acid) radical scavenging rate
DPPH•	2,2-Diphenyl-1-picrylhydrazyl radical scavenging rate
•OH	Hydroxyl free radical scavenging capacity
UPLC-MS/MS	Ultra-high-performance liquid chromatography–tandem mass spectrometry
LC-MS/MS	High-performance liquid chromatography–tandem mass spectrometry
MRM	Multiple reaction monitoring
PCA	Principal component analysis
PC1	Principal Component 1
PC2	Principal Component 2
OPLS-DA	Orthogonal partial least-squares discriminant analysis
TIC	Total ion current chromatogram
TICs	Total ion current chromatograms
QC	Quality control
ESI+	Positive electrospray ionization
ESI−	Negative electrospray ionization
Q^2^	Model predictability
R^2^	Model interpretability
R^2^Y	Model (for Y-variable dataset) interpretability
R^2^X	Model (for X-variable dataset) interpretability
DAMs	Differential accumulated metabolites
HCA	Hierarchical cluster analysis
ROS	Reactive oxygen species
