# Supplementary figures and images for: Effects of Different Temperatures on Flavonoid Secondary Metabolites and Antioxidant Activities of Paecilomyces hepiali
Source: Antioxidants (Basel). 2026 Jul 19;15(7):892. doi: 10.3390/antiox15070892 (PMC13404621; doi:10.3390/antiox15070892)

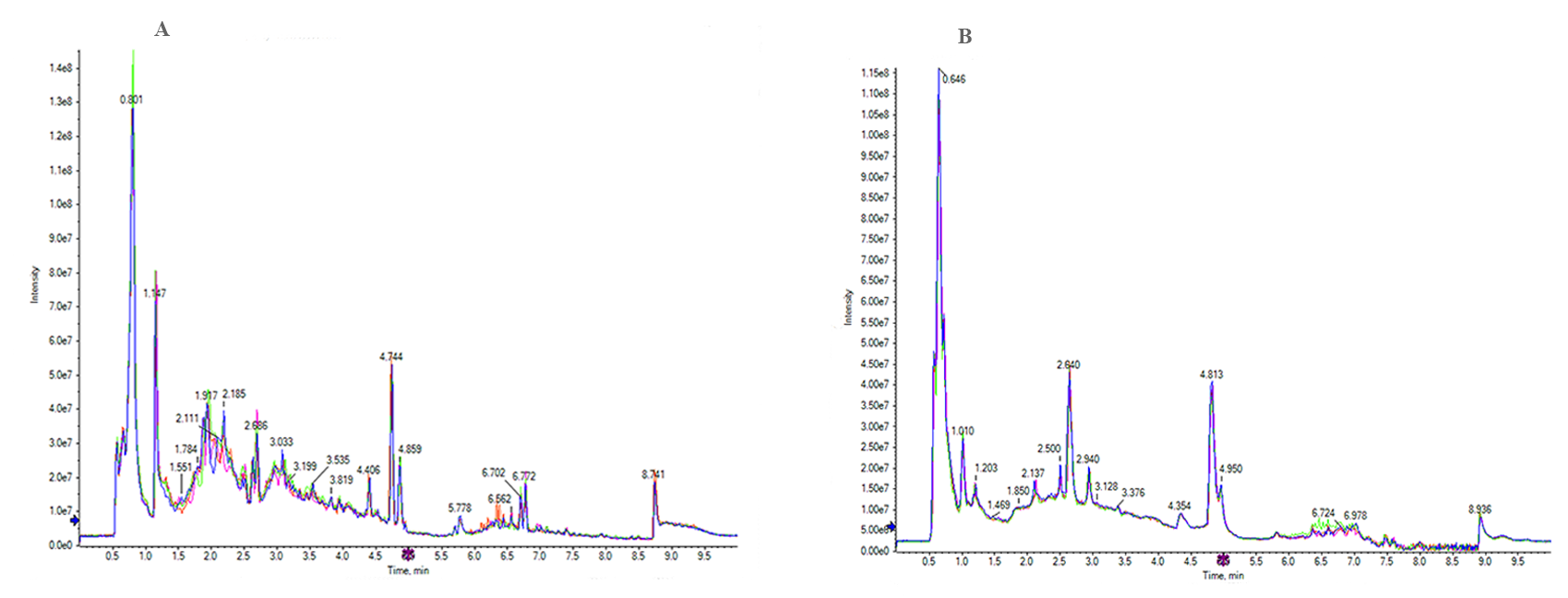

Supplement: Supplementary file 1 [file antioxidants-15-00892-s001.zip › Figure S1.png]

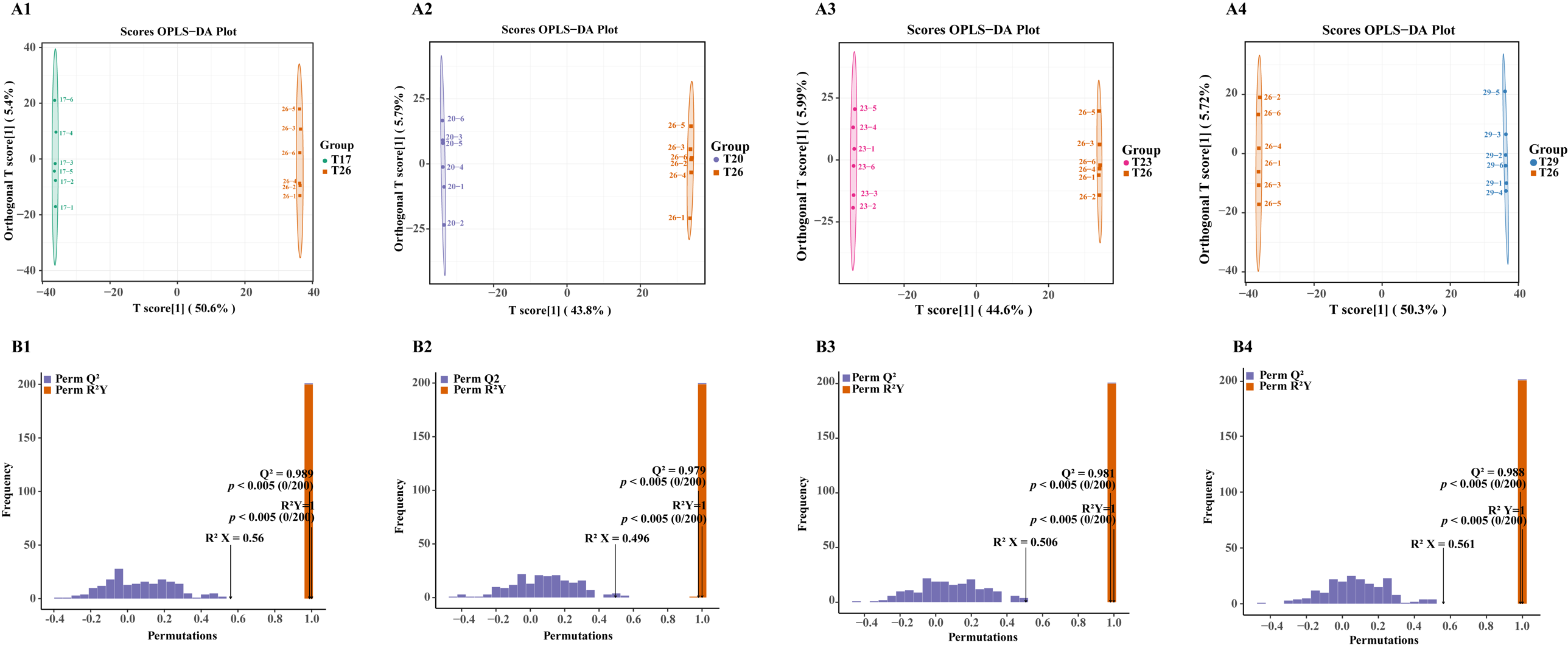

Supplement: Supplementary file 1 [file antioxidants-15-00892-s001.zip › Figure S2.png]
